# Perioperative nursing process-oriented bundled interventions and digital monitoring for the prevention of surgical site infections

**DOI:** 10.3389/fmed.2026.1837050

**Published:** 2026-06-09

**Authors:** Yingcheng Yang, Shifu Yao, Mingyu Pu, Xiaoqin Zhao, Yan Li, Ziqin Shu, Liu Xu, Lin Kang, Zhaoxia He

**Affiliations:** 1 Operating Room Nursing Unit, Department of Nursing, Chengdu Wenjiang District People’s Hospital, Chengdu, China; 2 Department of Anesthesiology, Chengdu Wenjiang District People’s Hospital, Chengdu, China; 3 Chengdu Wenjiang District People’s Hospital, Chengdu, China

**Keywords:** bundled interventions, digital surveillance, nursing, perioperative care, surgical site infection

## Abstract

Surgical site infection (SSI) remains a common healthcare-associated infection and is associated with prolonged hospitalization, readmission, increased costs, and adverse clinical outcomes. Because SSI prevention, detection, and management extend across the preoperative, intraoperative, postoperative, and post-discharge phases, SSI can be regarded as a perioperative outcome closely related to nursing assessment, surveillance, education, and continuity of care. This narrative review summarizes current evidence on perioperative SSI prevention from a nursing perspective, with emphasis on nursing process-oriented bundled interventions, digital surveillance, risk-stratified care, and nurse-led implementation strategies. Current evidence suggests that SSI prevention should move beyond isolated measures and be embedded into a continuous nursing pathway. Preoperative care should focus on risk identification, preparation verification, checklist-based checkpoints, and patient education. Intraoperative nursing should emphasize aseptic coordination, environmental control, physiological support, and actionable handover. Postoperative and post-discharge care should strengthen structured wound assessment, incision care, remote follow-up, and timely escalation of abnormalities. Digital tools, including electronic health record-based screening, remote wound monitoring, artificial intelligence-assisted image triage, and digital risk stratification, may extend nursing surveillance, but their effectiveness depends on clearly assigned responsibilities, predefined response timelines, workflow integration, and quality indicators. Nurse-led standardized pathways, stratified education, team training, and audit-feedback mechanisms are essential for translating evidence-based SSI prevention into stable clinical practice. Future research should further evaluate implementation quality, surveillance standardization, external validation of digital tools, cost-effectiveness, patient experience, and multidisciplinary sustainability.

## Graphical abstract

**Figure:**
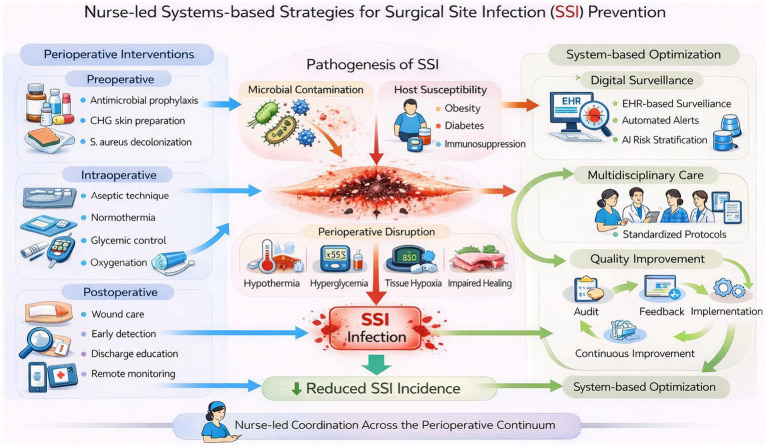

## 1 Introduction

Surgical site infection (SSI) remains one of the most common and burdensome healthcare-associated infections worldwide. A recent systematic review reported that the pooled global incidence of SSI was approximately 2.5% (95% CI, 1.6–3.7), with substantial variation across regions, surgical procedures, and patient populations (1). This burden is particularly pronounced in low- and middle-income countries, where limited perioperative care capacity may further increase the risk of infection. Routine European surveillance data from 2021 to 2022 showed that SSI incidence ranged from 0.6% for laminectomy to 9.6% for open colon surgery, highlighting marked differences across procedure types. Compared with patients without SSI, those who develop SSI experience longer hospital stays, higher readmission rates, greater healthcare costs, and increased mortality risk. Large-scale studies from high-income countries have similarly estimated that the incidence of SSI among hospitalized surgical patients is approximately 0.5 to 3% (2). Together, these findings indicate that SSI continues to impose a substantial burden on both patients and healthcare systems.

From a nursing perspective, SSI prevention depends on coordinated care throughout the entire perioperative period rather than on any single measure. Nurses play a central role in preoperative risk assessment and preparation, support for intraoperative infection prevention practices, postoperative wound observation, patient education, and post-discharge follow-up. Therefore, SSI is not only an issue of infection control, but also a perioperative outcome closely related to nursing assessment, nursing surveillance, and continuity of care. Current practice increasingly emphasizes bundled management, in which a small number of key evidence-based measures are implemented in a standardized manner. Core components commonly include timely antimicrobial prophylaxis, strict adherence to aseptic technique, maintenance of normothermia and glycemic control, and standardized wound care (3). Compared with isolated interventions, bundled strategies are more compatible with routine nursing workflows and are more conducive to process monitoring and quality improvement.

In recent years, digital tools have further expanded the role of nursing in SSI prevention and surveillance. Automated or semi-automated surveillance systems embedded in the electronic health record (EHR) can reduce manual workload and improve consistency in case identification (4). Postoperative remote wound follow-up, particularly when combined with image-based assessment and artificial intelligence-assisted interpretation, may facilitate earlier detection of post-discharge infection (5). In addition, machine learning models based on EHR data have shown moderate to high performance in predicting SSI risk and may support risk stratification and more efficient allocation of nursing resources (6). However, whether these tools can truly improve outcomes still depends on whether they are effectively integrated into existing nursing workflows and linked to clear responsibilities, response pathways, and continuous quality improvement mechanisms.

Against this background, this review examines SSI prevention and management from a nursing perspective, with a particular focus on perioperative nursing process-oriented bundled interventions, digital surveillance, risk-stratified nursing care, and nurse-led implementation and quality improvement strategies. The aim of this review is to synthesize current evidence, clarify how nursing can support SSI prevention throughout the preoperative, intraoperative, postoperative, and post-discharge phases, and explore how implementation quality and continuity of care can be improved through standardized pathways, multidisciplinary collaboration, and continuous evaluation.

This article adopts a narrative review approach. The search primarily covered literature published from January 2010 to April 2026, while earlier landmark guidelines or foundational studies were included when necessary. English-language literature related to surgical site infection, nursing, perioperative care, bundled interventions, wound surveillance, digital health, and remote follow-up was searched in PubMed, Web of Science, and Embase, with supplementary screening of relevant guidelines, consensus statements, and reference lists. The included literature mainly addressed perioperative SSI prevention, nursing practice, continuity of surveillance, digital support tools, risk-stratified nursing care, and implementation management. As the purpose of this review was to integrate and discuss existing evidence, a narrative synthesis was conducted rather than a quantitative analysis.

## 2 Nursing-related risk factors, infection progression, and perioperative significance of SSI

### 2.1 Perioperative burden of SSI and its relevance to nursing care

SSI remains one of the most common healthcare-associated infections and continues to impose a substantial burden on surgical patients and healthcare systems. Its incidence varies across countries, institutions, procedure types, and surveillance methods. In low- and middle-income countries, reported SSI rates commonly range from 8 to 30%, while large-scale studies and surveillance data from high-income settings also show marked procedure-related heterogeneity, with relatively high rates after open abdominal procedures and in selected orthopedic populations (2, 7–10). SSI is associated with prolonged hospitalization, increased healthcare costs, higher readmission rates, postoperative complications, and increased mortality risk; in severe cases, it may progress to sepsis and require intensive care support (2, 7).

According to infection depth, SSI is commonly classified as superficial incisional, deep incisional, or organ/space infection (11). This classification is clinically important because it influences wound assessment priorities, follow-up intensity, escalation thresholds, and treatment pathways. From a nursing perspective, SSI is not merely an infection control outcome, but a perioperative event closely related to assessment quality, process stability, surveillance continuity, and patient education. Preoperative preparation, intraoperative aseptic coordination, postoperative wound observation, and post-discharge follow-up all affect the prevention, detection, and management of SSI (3, 12, 13). Therefore, SSI can be regarded as a nursing-sensitive perioperative outcome that reflects the continuity and reliability of care across the surgical pathway.

### 2.2 Risk factors that should be prioritized in nursing assessment

SSI is a multifactorial complication caused by interactions among patient susceptibility, surgical exposure, microbial burden, and care-system factors. For nursing practice, the purpose of risk factor assessment is not only to identify high-risk patients, but also to guide the intensity of preoperative preparation, postoperative observation, discharge education, and follow-up planning.

Patient-level risk factors are among the most consistently reported predictors of SSI. Older age, obesity, diabetes, smoking, malnutrition, corticosteroid exposure, immunosuppression, and human immunodeficiency virus infection have been associated with increased SSI risk (14–16). These conditions may impair tissue perfusion, delay wound healing, alter immune defense, or reduce physiological reserve. In some patients, combined risk factors, such as elevated body mass index and immunosuppression, may further increase vulnerability (16, 17). For nurses, these factors should be translated into practical interventions, including nutritional screening, glycemic evaluation, smoking cessation education, assessment of self-care capacity, and targeted postoperative monitoring.

Surgery-related and system-level factors are also important. Open procedures, prolonged operative duration, contaminated incisions, severe tissue injury, prosthetic implantation, and technically complex operations are associated with a higher risk of SSI (14, 17–19). Care-system factors, including prolonged hospitalization, surveillance quality, local infection control capacity, and perioperative documentation, may further influence both infection occurrence and case detection. Previous infection history, incision classification, and other perioperative variables have also been incorporated into electronic health record-based or machine learning-based SSI prediction models, indicating that risk assessment is increasingly moving toward structured and data-supported stratification (6, 20). From a nursing perspective, such risk information should be used to identify which patients require intensified education, closer wound assessment, earlier reassessment, or more frequent post-discharge contact.

### 2.3 Major pathways of SSI development and nursing-relevant perioperative nodes

**Figure 1:**
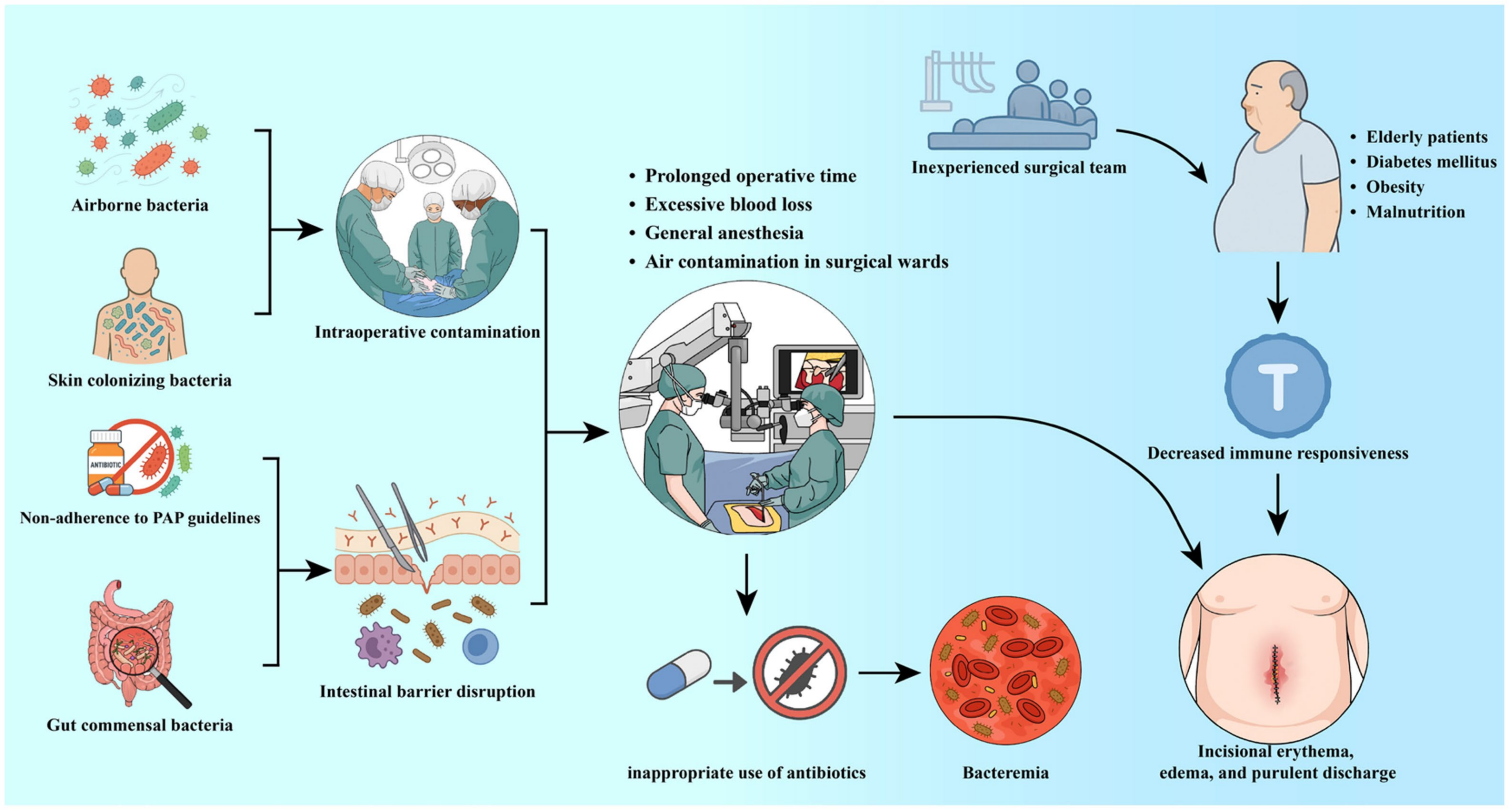
Major pathways of surgical site infection development and nursing-relevant perioperative nodes. This figure illustrates the main mechanisms contributing to surgical site infection, including microbial contamination, host susceptibility, intraoperative exposure, and failures in preventive processes, and highlights the perioperative nodes at which nursing care may influence prevention, early recognition, and escalation.

SSI develops through the combined effects of microbial contamination, host susceptibility, surgical exposure, and failures in preventive processes. Understanding these pathways is important for nursing practice because it helps identify the perioperative nodes where nursing actions can reduce risk or support early recognition. The major pathways of SSI development and nursing-relevant perioperative nodes are summarized in Figure 1.

First, microbial exposure may occur through skin commensals, endogenous patient flora, contaminated instruments, healthcare personnel contact, or environmental sources (21–25). In orthopedic and implant-related surgery, *Staphylococcus aureus* colonization is a major risk factor, whereas abdominal and gastrointestinal procedures are more often associated with enteric organisms and mixed aerobic–anaerobic flora (21–23). These findings suggest that preoperative decolonization, skin preparation, intraoperative aseptic support, and environmental discipline should be understood as linked interventions across the same infection pathway rather than as isolated tasks.

Second, whether contamination progresses to infection depends strongly on host defense capacity. Diabetes, obesity, malnutrition, impaired immune function, tissue ischemia, and poor wound-healing ability can reduce resistance to microbial invasion. In abdominal surgery, mucosal barrier disruption and intestinal manipulation may further increase exposure to enteric pathogens (22, 23, 26–28). For nursing care, this means that patients undergoing similar procedures may require different levels of surveillance depending on their physiological reserve and wound-healing capacity. Thus, procedure-based care should be combined with patient-level vulnerability assessment.

Third, SSI risk increases when preventive measures are not implemented accurately, timely, or consistently. Inappropriate antimicrobial prophylaxis timing, suboptimal antibiotic selection, unnecessary prolongation of prophylaxis, aseptic breaches, fragmented wound surveillance, and delayed escalation of abnormal findings may all weaken prevention (3, 29–32). This indicates that SSI is not only a postoperative complication, but also the cumulative result of risk across preoperative, intraoperative, postoperative, and post-discharge stages. For nurses, preoperative verification, intraoperative deviation recognition, structured wound assessment, and remote follow-up should therefore be connected within a continuous surveillance pathway.

Foreign materials, including implants, sutures, and catheters, may further promote bacterial adhesion and biofilm formation, increasing the risk of persistent infection and complicating treatment (33–36). Antimicrobial resistance and limited microbiological diagnostic capacity may also delay pathogen identification and appropriate management (33–35). Therefore, in implant-related or high-risk procedures, even mild early wound changes should be interpreted cautiously. Nursing observation should focus not only on overt infection signs, but also on whether wound healing deviates from the expected course and whether timely clinical reassessment or specimen collection is required.

### 2.4 Implications for perioperative nursing practice

Taken together, SSI should be viewed as the result of patient susceptibility, surgical exposure, microbial contamination, and the quality of perioperative prevention and surveillance. This understanding has two implications for nursing practice. First, risk assessment should be translated into stratified nursing management, including tailored preoperative preparation, intraoperative communication, postoperative observation frequency, discharge education, and follow-up intensity. Second, SSI prevention should rely on continuous nursing care rather than isolated measures. Evidence-based interventions become clinically meaningful only when they are embedded into standardized pathways, supported by structured documentation, and linked to early recognition and escalation of abnormal healing (3, 31, 32, 37).

Accordingly, this section clarifies why perioperative nursing bundled interventions are necessary. By linking risk identification, infection progression, and nursing action, perioperative care can move from fragmented task execution toward a more targeted, risk-stratified, and continuous prevention and surveillance model.

## 3 Perioperative nursing process-oriented bundled interventions for SSI prevention

Perioperative SSI prevention should be understood as a continuous nursing process rather than as the simple accumulation of isolated preventive measures. Although current guidelines have clearly defined many core components of SSI prevention, including skin preparation, antimicrobial prophylaxis, aseptic practice, normothermia, glycemic control, wound care, and post-discharge follow-up, the practical challenge is how to embed these measures into a consistent workflow across different perioperative stages. From a nursing perspective, the value of bundled intervention lies not in repeating guideline recommendations, but in transforming them into risk-based assessment, verifiable checkpoints, structured handover, continuous wound surveillance, and timely escalation.

**Figure 2:**
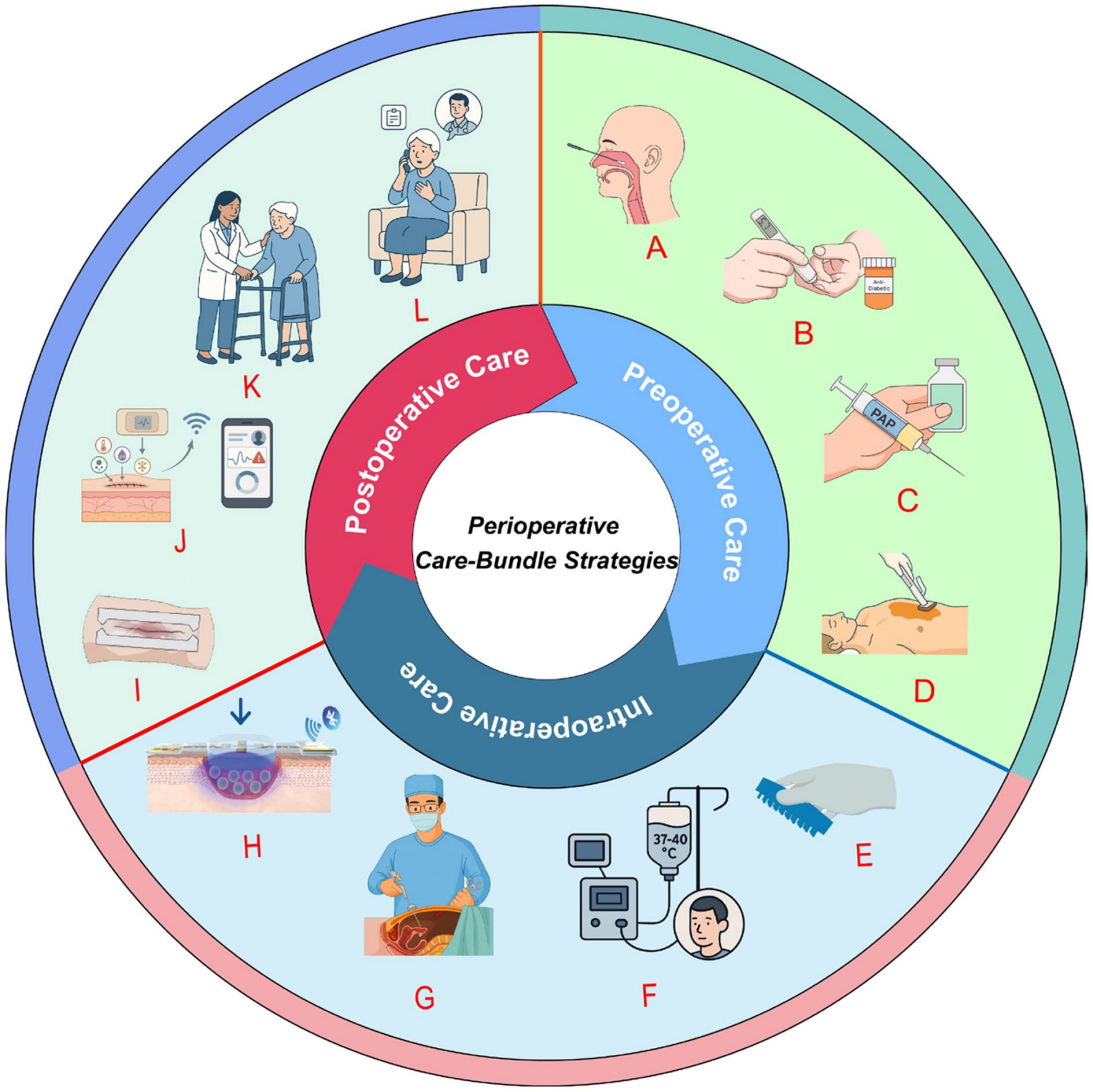
Perioperative nursing care-bundle strategies for surgical site infection prevention. This figure summarizes process-oriented bundled nursing interventions across the preoperative, intraoperative, postoperative, and post-discharge phases, emphasizing continuity of surveillance, patient education, handoff, and timely response to abnormalities.

Accordingly, this section focuses on how nursing care links preoperative risk identification, intraoperative process control, postoperative wound assessment, and post-discharge monitoring into an integrated pathway. The integrated perioperative nursing pathway is summarized in Figure 2. This pathway also provides the operational basis for digital nursing surveillance, because electronic checklists, EHR-based risk screening, remote wound image review, and AI-assisted alerts can only be effective when they are connected to clearly defined nursing responsibilities and response procedures.

### 3.1 Preoperative phase: risk identification and checkpoint verification

The preoperative phase should primarily serve as the entry point for risk stratification and preparation verification. Instead of repeating all preventive measures in detail, nursing assessment should focus on identifying patients who require intensified intervention, including those with advanced age, diabetes, obesity, malnutrition, immunosuppression, smoking history, poor self-care ability, previous infection, local skin abnormalities, or procedures involving implants (3, 14–17). These factors should be translated into practical nursing decisions, such as whether the patient requires additional education, caregiver involvement, closer postoperative wound observation, or earlier post-discharge follow-up.

Core preoperative measures, including bathing or skin cleansing, appropriate hair management, allergy confirmation, antimicrobial prophylaxis timing, and targeted decolonization when indicated, should be incorporated into a standardized checklist rather than discussed as independent guideline items (16, 17, 31, 38–41). The nursing role is to verify whether these measures have been completed, document unresolved issues, and communicate high-risk information before the patient enters the operating room (3, 42). For older patients or those with limited mobility, cognitive impairment, or weak social support, education should be simplified, repeated, and, when appropriate, delivered together with caregivers (32, 42).

Thus, the key output of the preoperative phase is not only completion of preparation, but also the formation of a clear risk profile. This profile should be available for intraoperative and postoperative teams and, ideally, should be embedded in the electronic perioperative record to support digital risk flagging and follow-up planning. A proposed risk-stratified nursing protocol is provided in Table 1.

**Table 1:** Proposed risk-stratified nursing protocol for SSI prevention.

Risk level	Suggested criteria	Nursing intervention
Low risk	Clean incision, no major comorbidity, good self-care ability	Standard checklist, routine wound education, scheduled follow-up
Moderate risk	Diabetes, obesity, smoking, malnutrition, open surgery, prolonged operation, limited health literacy	Reinforced education, caregiver involvement if needed, structured wound assessment, 48–72 h telephone/app follow-up
High risk	Immunosuppression, contaminated wound, implant-related surgery, previous infection, poor self-care, cognitive impairment, weak social support	Wound care nurse review, discharge photo-upload training, 24–48 h active follow-up, lower escalation threshold

The proposed follow-up intervals are intended as practical examples and should be adapted according to surgical specialty, institutional resources, patient condition, and local SSI surveillance policies.

### 3.2 Intraoperative phase: process stability and actionable handover

The intraoperative phase should focus on maintaining process stability and preventing deviations from being carried forward into postoperative care. Nurses support SSI prevention by monitoring aseptic practice, coordinating workflow continuity, reducing unnecessary environmental disruption, and confirming that key physiological targets such as normothermia and glycemic control are addressed when clinically relevant (3, 38, 43–46). These elements are already well established in guidelines; therefore, the emphasis here should be on nursing surveillance, deviation recognition, and timely correction.

In practice, circulating and scrub nurses are positioned to detect contamination events, glove or drape problems, breaks in sterile technique, delays in workflow, and other events that may increase infection risk. Such events should not remain informal observations. They should be documented, communicated, and converted into postoperative surveillance priorities. For example, prolonged operative duration, implant use, intraoperative contamination, abnormal temperature or glucose control, and antimicrobial redosing should be included in handover because they may influence the frequency and focus of postoperative wound assessment.

Digital tools may strengthen this process by embedding intraoperative checkpoints into electronic checklists and by generating structured handover prompts. However, these tools should support, rather than replace, nursing judgment. Their value depends on whether the system can capture clinically meaningful deviations and whether specific staff members are responsible for reviewing and acting on these signals.

### 3.3 Postoperative and post-discharge phases: structured surveillance and digital continuity

The postoperative and post-discharge phases are the most important stages for detecting evolving SSI. Many infections are not apparent immediately after surgery and may emerge during ward observation or after discharge (4, 5, 47, 48). Therefore, postoperative nursing should shift from routine dressing management to structured surveillance based on wound appearance, symptom progression, patient-reported changes, and risk level.

Ward-based wound assessment should follow a structured and trend-oriented format, including local inflammatory signs, exudate characteristics, odor, wound dehiscence, fever, pain trajectory, and delayed recovery progress (3, 47). In high-risk patients, surveillance should be intensified through more frequent assessment and lower thresholds for escalation, with abnormal findings directed to predefined clinical review pathways rather than managed only by routine dressing care (3, 5, 49). Dressing changes should be performed according to wound condition and clinical indication, while adjunctive measures should be selected according to patient risk and incision characteristics: closed-incision negative pressure therapy may be considered for selected high-risk incisions (39, 50–54), antimicrobial dressings may be used as supportive options in wounds with increased contamination or infection concern (55–58), and smart wound technologies remain emerging tools for early abnormality detection rather than routine replacements for nursing assessment (59–61). Therefore, the central nursing task is to document wound trajectories, identify deviations from expected healing, and initiate timely reassessment within an established escalation pathway (3, 47).

Post-discharge follow-up should be treated as a continuation of inpatient surveillance. Telephone review, structured symptom questionnaires, patient-uploaded wound photographs, and remote wound monitoring can extend nursing observation beyond hospitalization (5, 48, 49). However, these approaches are useful only when workflow details are explicit: who reviews the submitted information, how quickly alerts should be checked, which findings require clinician reassessment, and how patients should be instructed to seek help. For elderly patients or those with limited digital literacy, nurses should provide hands-on education before discharge, including how to photograph the wound, when to upload images, which symptoms to report, and whom to contact if the system cannot be used.

This phase also forms the direct bridge between bundled nursing care and AI-supported digital monitoring. AI-based wound assessment or electronic alerts should be positioned as triage-support tools that help prioritize review, not as autonomous diagnostic systems. When suspicious abnormalities are detected, the response pathway should move from digital signal to nursing review, clinical reassessment, microbiological evaluation when needed, and treatment decision. Without this closed-loop process, digital monitoring may increase information burden without improving patient outcomes.

### 3.4 Summary of the nursing pathway

Overall, perioperative bundled nursing intervention should be reframed as a closed-loop pathway: preoperative risk stratification, intraoperative deviation capture, postoperative structured wound assessment, and post-discharge digital surveillance. This structure avoids unnecessary repetition of established guideline content and highlights the author’s main synthesis: digital technologies and AI can extend nursing surveillance only when they are embedded into defined workflows, assigned to responsible personnel, linked to response timelines, and evaluated through nursing quality indicators.

## 4 Implementation of digital nursing surveillance in clinical workflows

Digital surveillance should be understood as an operational extension of perioperative nursing rather than as a technical layer added outside routine care. In SSI prevention, EHR-based screening, remote wound follow-up, AI-assisted image assessment, electronic checklists, and machine learning-based risk stratification can support nursing assessment, surveillance continuity, early warning, and post-discharge follow-up. However, their clinical value depends less on technical availability alone than on whether they are embedded into clearly defined workflows, assigned to responsible staff, linked to response timelines, and evaluated through quality indicators. Therefore, this section focuses on how digital tools can be implemented within clinical nursing practice, with particular attention to alert monitoring, response pathways, patient education, and organizational accountability. A practical implementation framework for digital nursing surveillance of SSI is summarized in Table 2.

**Table 2:** Implementation framework for digital nursing surveillance of SSI.

Digital component	Main information source/trigger	Responsible personnel	Nursing response and timeline	Escalation/quality indicator
EHR-based SSI screening	Microbiology results, antimicrobial use, fever records, readmission, operative data, wound documentation	Infection surveillance nurse or designated perioperative nurse	Verify alerts, review wound records, and contact the bedside nurse or patient when needed. High-risk alerts should receive same-day review; moderate-risk alerts may be reviewed within 24 h.	Escalate suspected SSI to the surgical or infection control team; monitor alert review rate and time to review.
Electronic perioperative checklist	Skin preparation, allergy history, antimicrobial prophylaxis timing, hair management, temperature- or glucose-related documentation	Circulating nurse, bedside nurse, perioperative coordinating nurse	Confirm key checkpoints before incision, during handover, and before discharge; document unresolved or incomplete items.	Monitor checklist completion rate, missing-item rate, and correction rate before incision or discharge.
Digital risk stratification	Patient comorbidities, procedure type, incision classification, implant use, previous infection, self-care capacity	Perioperative nurse, wound care nurse, nursing manager	Classify patient risk before surgery and update before discharge; use the risk level to determine education intensity and follow-up needs.	Monitor high-risk identification rate and follow-up completion rate.
Remote wound follow-up	Mobile app, structured symptom questionnaire, telephone follow-up, wound photographs	Wound care nurse or designated follow-up nurse	Review symptoms and wound images; request repeat images if unclear. High-risk patients may receive active follow-up within 24–48 h after discharge; moderate-risk patients may receive follow-up within 48–72 h.	Monitor photo submission rate, failed contact rate, and time from abnormal report to clinical review.
AI-assisted wound image triage	Patient-uploaded wound photographs and AI-generated risk output	Nurse reviewer with clinician support when needed	Check image quality, verify symptoms, and compare AI output with the expected healing trajectory. High-risk AI outputs should trigger same-day nursing verification.	Escalate discordant or high-risk cases; monitor false-positive and false-negative cases.
Elderly patient digital education	Discharge teaching, supervised app trial, caregiver participation	Bedside nurse or discharge nurse	Demonstrate app use, photo capture, symptom reporting, and emergency contact pathways before discharge; involve caregivers when independent app use is difficult.	Monitor successful trial upload rate and caregiver participation rate.
Closed-loop escalation	Digital alert, wound image, symptom report, nurse assessment	Nurse reviewer, surgeon, infection control team	Document abnormalities and initiate reassessment. Urgent signs should receive same-day review; non-urgent abnormalities may be reviewed within 24–48 h.	Monitor unresolved alert rate, escalation completion rate, and wound-related readmission.

The proposed response timelines are intended as an implementation framework and should be adapted according to institutional resources, patient risk, and local SSI surveillance policies.

### 4.1 EHR-embedded surveillance and alert monitoring

EHR-embedded surveillance systems can identify potential SSI events by integrating microbiological results, antimicrobial prescriptions, fever records, readmission signals, procedure-related variables, and clinical documentation (4, 47). Semi-automated algorithms have shown potential to improve case detection and reduce manual workload, especially when they are externally validated and aligned with standardized surveillance definitions (62, 63). Nevertheless, an automated alert itself does not constitute clinical surveillance. Its value depends on whether the alert is reviewed, verified, documented, and acted upon within a defined nursing pathway.

In practice, institutions may establish a tiered alert-monitoring workflow based on EHR-embedded SSI surveillance and locally validated screening criteria. Low-risk reminders may be incorporated into routine ward review or scheduled post-discharge follow-up. Moderate-risk alerts, such as mild wound symptoms, delayed recovery, or incomplete follow-up information, should be reviewed by a designated perioperative nurse, wound care nurse, or infection surveillance nurse within a defined timeframe (3, 5, 49). High-risk alerts, such as positive wound cultures, readmission with suspected infection, persistent fever, purulent drainage, wound dehiscence, or rapidly worsening pain, should trigger same-day nursing verification and escalation to the surgical team or infection control team.

The responsibility for alert review should be explicitly assigned, because automated surveillance is effective only when digital signals are linked to manual verification, clinical reassessment, and defined response pathways (4, 5). Bedside nurses may conduct initial wound assessment and documentation during hospitalization. A wound care nurse or infection surveillance nurse may verify alerts and coordinate reassessment. Surgeons or infection specialists should make diagnostic and treatment decisions when SSI is suspected. Information technology staff should maintain data extraction, alert logic, and platform function, but should not be responsible for clinical interpretation. This division of responsibility prevents digital surveillance from becoming a passive data-generation system without clinical action (49).

### 4.2 Remote wound follow-up and patient-submitted wound images

Because a substantial proportion of SSIs emerge after discharge, remote wound follow-up is an important component of digital nursing surveillance. Mobile applications, structured symptom questionnaires, patient-uploaded wound photographs, and telephone follow-up may help identify suspicious wound changes before routine outpatient visits or readmission (5, 48, 49). However, remote monitoring is effective only when patients know what information to submit, nurses have protected time to review submissions, and abnormal findings are linked to timely clinical reassessment (49).

A practical remote wound follow-up protocol should specify the timing, content, reviewer, and escalation threshold. Patient-generated wound photographs and symptom reports have been used in smartphone-based postoperative wound monitoring and may support earlier triage and clinical advice when connected to a defined review pathway (64–67). For low-risk patients, scheduled symptom review may be sufficient. For moderate-risk patients, telephone or app-based follow-up within 48–72 h after discharge may be considered where resources allow. For high-risk patients, active follow-up within 24–48 h may be appropriate, but the exact timeline should be adapted to institutional capacity and local SSI surveillance policies. Submitted information should include wound photographs and key symptoms such as fever, increasing pain, redness, swelling, exudate, odor, wound separation, and difficulty with wound care (64–67).

Education on wound image submission is particularly important for older patients and those with limited digital literacy. Before discharge, nurses should demonstrate app use, wound photography, symptom questionnaire completion, and information submission; when possible, patients or caregivers should complete one supervised trial upload (66, 68, 69). Image instructions should emphasize adequate lighting, complete visualization of the incision and surrounding skin, and avoidance of blurred or shadowed images (70, 71). If independent app use is not feasible, telephone follow-up, caregiver-assisted submission, secure message-based photo upload, or earlier outpatient review should be arranged.

### 4.3 AI-assisted wound assessment as a triage-support tool

AI-assisted image interpretation and computer vision tools may further support postoperative wound surveillance by helping identify abnormal wound features and prioritize cases for review (5, 72–74). These tools may reduce part of the manual screening burden and improve the consistency of preliminary triage. However, current models remain dependent on image quality, patient population, wound type, and external validation. Therefore, AI-assisted assessment should be positioned as a triage-support tool rather than an autonomous diagnostic system (73, 74).

In clinical workflow, AI outputs should be reviewed together with patient-reported symptoms and nursing assessment, because current image-based AI models for surgical wound infection detection remain dependent on image quality, case mix, model validation, and clinical context (75). For example, an AI-generated high-risk wound image alert should prompt a nurse to verify the photograph, contact the patient if necessary, review symptoms, and determine whether clinical reassessment is required. Low-quality or unclear images should not be interpreted as reassuring; instead, the patient or caregiver should be asked to resubmit the image or receive telephone review, because standardized wound photography methods are necessary to support reliable remote SSI assessment (70, 71). When AI assessment conflicts with clinical symptoms, nursing and clinician judgment should take priority, consistent with current recommendations that clinical AI should support, rather than replace, human decision-making (76–79).

The implementation of AI-assisted wound assessment also requires explicit accountability. Institutions should define who reviews AI alerts, how false positives and false negatives are audited, how model performance is monitored over time, and when the system should be recalibrated or suspended. These requirements are consistent with broader recommendations for clinical AI evaluation and governance, which emphasize human factors, safety monitoring, transparent reporting, post-deployment performance review, and accountability across the AI lifecycle (78, 79). Without these safeguards, AI tools may increase alert burden or create inappropriate reassurance. Their role should therefore be limited to improving review priority, supporting continuity of surveillance, and assisting early recognition, while final clinical decisions remain the responsibility of trained healthcare professionals (76, 77).

### 4.4 Digital risk stratification and electronic checklists

Digital tools can also support SSI prevention by converting patient- and procedure-related risk factors into actionable nursing plans. Machine learning models based on structured EHR data have shown moderate to good performance in predicting SSI risk or supporting case detection (6, 80, 81). In nursing practice, the main value of risk prediction is not the score itself, but its ability to guide resource allocation. For example, patients classified as high risk may receive intensified discharge education, earlier post-discharge contact, wound care nurse review, caregiver involvement, and lower thresholds for clinical reassessment.

Electronic checklists may improve the reliability of perioperative workflows by prompting completion of time-sensitive tasks, such as antimicrobial prophylaxis verification, allergy confirmation, skin preparation documentation, hair management, temperature-related monitoring, and structured handover (82, 83). Computerized antimicrobial stewardship tools may also support appropriate prophylactic use through reminders, prescribing support, and feedback (84, 85). However, checklist completion should not become a purely administrative action. For digital checklists to improve SSI prevention, each checkpoint should be linked to a responsible person, a required action, and a response pathway when the item is incomplete or abnormal.

For example, if antimicrobial prophylaxis timing is not documented before incision, the system should not simply record missing data; it should prompt verification by the circulating nurse and communication with the anesthesia or surgical team, consistent with the checklist principle that time-sensitive safety items should be confirmed before incision (86). If a high-risk wound is identified at discharge, the system should automatically generate a follow-up task and assign it to a designated nurse. In this way, electronic checklists function not only as documentation tools, but also as workflow-control tools that improve checklist adherence and reduce omission of key perioperative steps (87).

### 4.5 Closed-loop workflow and organizational accountability

The implementation of digital SSI surveillance should follow a closed-loop pathway: risk identification, digital alert generation, nursing review, clinical verification, escalation, intervention, documentation, and audit-feedback. Each step should have an accountable role. Bedside nurses are responsible for structured wound assessment, patient education, and documentation. Wound care nurses or infection surveillance nurses are responsible for reviewing alerts, verifying submitted wound images, and coordinating follow-up. Surgeons and infection control specialists are responsible for diagnostic evaluation and treatment decisions. Nursing managers and quality improvement personnel are responsible for monitoring response indicators and correcting workflow failures. Information technology teams are responsible for system stability, data integration, and alert maintenance (79, 88).

Quality indicators should be used to determine whether digital monitoring is functioning as intended. These may include the proportion of alerts reviewed within the required timeframe, the median time from alert generation to nursing review, the median time from abnormal finding to clinical reassessment, completion rate of post-discharge wound photo submission, follow-up completion rate among high-risk patients, proportion of unresolved alerts, false-positive and false-negative alert review, wound-related readmission rate, and standardized SSI rate. Regular audit and feedback can help identify whether failures occur at the level of patient education, alert review, escalation, clinician reassessment, or documentation (88, 89).

Overall, the core value of digital nursing surveillance lies in transforming fragmented postoperative information into a timely and accountable clinical response. EHR screening, AI-assisted assessment, mobile wound follow-up, and electronic checklists should not be implemented as independent technologies. They should be embedded into a nurse-led, multidisciplinary workflow in which alerts are monitored by designated staff, patients are educated to submit usable information, response timelines are predefined, and organizational accountability is maintained through quality indicators (79, 89).

## 5 Nurse-led implementation pathways, stratified nursing care, and continuous quality improvement

Building on the digital workflow described above, this section discusses how nurse-led pathway governance, stratified education, team training, and audit-feedback mechanisms can sustain SSI prevention and quality improvement in routine practice.

### 5.1 Standardized pathways and nurse-led governance

Evidence-based interventions can reduce SSI rates consistently only when they are translated into routine clinical practice through structured implementation. The role of a standardized pathway is to integrate the preoperative, intraoperative, postoperative, and post-discharge phases into a continuous care process. Pathway components may include verification of skin preparation and antimicrobial prophylaxis, temperature and glycemic targets, wound assessment standards, thresholds for responding to abnormal incision signs, and follow-up time points (3). The effectiveness of such pathways depends not only on whether SSI rates decline, but also on whether key measures are implemented consistently and reliably across different care settings.

From the perspective of the nursing process, nurse-led pathway governance aims to maintain continuity across assessment, implementation, surveillance, handover, and intervention. The value of this division of responsibilities lies not only in clarifying role-specific tasks, but also in ensuring that key preventive steps are not missed during transitions between care settings.

Standardized pathways also help reduce unnecessary variation in clinical practice and improve the recognition of deviations. Therefore, a nurse-led framework does not mean that a single role undertakes all tasks; rather, it means establishing clear collaborative relationships under a shared nursing goal in order to strengthen continuity across prevention, surveillance, and early intervention.

### 5.2 Individualized care and patient education based on risk stratification

Although standardized pathways provide the foundation for SSI prevention, care still needs to be adjusted according to patient risk. Individualized nursing care begins with risk stratification, that is, identifying patients with diabetes, malnutrition, obesity, immunosuppression, reduced self-care capacity, or inadequate conditions for out-of-hospital monitoring, and linking these risks to intensified perioperative support and follow-up arrangements (14–17, 90). The purpose of risk stratification is to adjust the intensity and mode of education, wound assessment, and follow-up according to patient vulnerability and self-care capacity.

Patient education should be embedded within this stratified care process. Standardized educational materials, checklists, and self-monitoring tools may help improve patient adherence to preoperative bathing, wound care, symptom recognition, and timely help-seeking (32). However, education should not be limited to a one-time transfer of information, but should be implemented in conjunction with wound surveillance, recognition of abnormalities, and pathways for obtaining assistance. Nursing staff need to adjust the content, mode of communication, and frequency of reinforcement according to the patient’s health literacy, communication ability, self-care capacity, and family support. For patients who may have difficulty recognizing or reporting warning signs in a timely manner, family members or primary caregivers should also be included in discharge preparation and follow-up planning.

Training of the nursing team is likewise a prerequisite for the stable implementation of individualized care. Systematic training on SSI definitions, risk factors, wound assessment, surveillance workflows, and response procedures can improve nurses’ knowledge and implementation capacity (91). Such training should be integrated as much as possible with actual surveillance tasks, case review, and feedback on workflow deficiencies, rather than being limited to one-time didactic sessions. This helps nursing staff apply SSI definitions more consistently, identify abnormal wound progression earlier, and take standardized action within established pathways. Therefore, individualized care requires not only adjustment of nursing intensity according to patient risk, but also a nursing team capable of sustaining stratified management in practice.

### 5.3 Nursing quality indicators, audit and feedback, and continuous quality improvement

Continuous quality improvement is an essential mechanism for sustaining the long-term operation of standardized pathways and individualized nursing care. Tools such as audit and feedback cycles, root cause analysis, and failure mode and effects analysis become truly valuable in practice only when infection prevention is treated as an ongoing organizational priority (92). From the perspective of nursing practice, the importance of quality improvement lies not only in observing whether infection rates change, but also in identifying where weaknesses occur in the nursing process and adjusting workflows accordingly to reduce recurrence of similar problems. Evaluation should combine process, outcome, implementation, and balancing indicators, but the selected indicators should be limited to those that can directly guide pathway correction and staff feedback. Through multidimensional evaluation, pathway performance is no longer reflected solely by infection rates.

Feedback of data should be timely, specific, and directly linked to retraining or workflow optimization. Concurrent review of process and outcome indicators is essential, because reduced adherence to bundled measures is associated with increased SSI risk, whereas higher adherence is generally associated with lower infection rates (93, 94). Accordingly, the role of audit is not merely retrospective reporting, but also identifying where breakdowns occur in verification, education, surveillance, handover, or management. Real-world studies suggest that, in certain settings including obstetric care, nurse-led bundled management combined with multidisciplinary collaboration and process monitoring can reduce SSI rates (95). Similarly, bundle-based quality improvement programs have shown potential to reduce SSI in selected settings, including obstetric care and gynecologic oncology (89, 95). Key breakpoints, nurse-led reconstruction priorities, and evaluation indicators across the perioperative SSI prevention pathway are summarized in Table 3.

**Table 3:** Key breakpoints, nurse-led reconstruction priorities, and evaluation focus across the perioperative SSI prevention pathway.

Stage	Key breakpoint	Nurse-led reconstruction priorities	Evaluation focus
Preoperative	High-risk patients are not clearly identified	Move risk assessment earlier; flag high-risk patients before surgery	High-risk identification rate
	Preoperative preparation is incomplete	Verify skin cleansing, skin preparation, antimicrobial prophylaxis, and decolonization	Completion rate of preoperative preparation
	Key information is not communicated in time	Standardize preoperative communication and handover	Completeness of preoperative handover
	Patient understanding is insufficient	Deliver stratified education; include caregivers when needed	Completion rate of preoperative education
Intraoperative	Aseptic breaches are not promptly recognized	Maintain continuous observation of the sterile field and workflow deviations	Documentation rate of aseptic deviations
	Intraoperative risk information is not transferred	Standardize recording of contamination, implant use, and abnormal events	Completeness of intraoperative handover
	Workflow instability weakens preventive measures	Strengthen coordination, equipment readiness, and interruption control	Adherence to key preventive measures
	Physiologic support is inconsistent	Reinforce temperature- and glucose-related monitoring and response	Normothermia rate; completion of glucose monitoring and response
Postoperative and post-discharge	Wound monitoring is fragmented	Use structured wound assessment and trend-based documentation	Completion rate of structured wound assessment
	Abnormal findings are recognized late	Define thresholds for reassessment and escalation	Time from abnormal finding to clinical review
	Post-discharge surveillance is weak	Integrate discharge education with scheduled follow-up	Follow-up completion rate
	Remote alerts do not trigger action	Set response time limits and assign responsible staff	Delayed response rate to alerts
Outcomes and balancing measures	Evaluation relies on infection rate alone	Review process, outcome, and balancing indicators together	Standardized SSI rate; wound-related readmission rate
	Intervention burden is not assessed	Monitor unnecessary antibiotic extension and dressing changes without indication	Inappropriate antibiotic extension rate; non-indicated dressing change rate

Overall, the implementation quality of perioperative SSI prevention depends on whether standardized pathways can be sustained with the support of stratified nursing care and quality feedback. In this process, the nursing team is responsible not only for implementing interventions, but also for identifying implementation deviations, promoting corrective actions, and translating surveillance data into safer routine practice.

## 6 Challenges, multidisciplinary collaboration, and future perspectives

Although the key principles of perioperative SSI prevention have become increasingly clear, current practice still faces several challenges. Differences remain across guidelines and original studies with respect to operational details, recommended thresholds, and the strength of evidence (3). Such variation may reduce consistency in frontline implementation and make it more difficult to compare outcomes across institutions. At the same time, the translation of digital tools into routine nursing practice remains incomplete. EHR-embedded automated or semi-automated surveillance systems may improve consistency in case identification and reduce manual workload (4, 47), while remote wound follow-up and AI-assisted assessment may support earlier recognition of suspected post-discharge infection (5, 72–74). However, these tools are likely to improve outcomes only when data quality is reliable, responsibilities are clearly defined, escalation pathways are explicit, and the tools are closely integrated into clinical workflows. For antimicrobial dressings, smart wound technologies, and some environmental interventions discussed above, current studies suggest a degree of potential utility, but sufficiently high-quality clinical evidence is still lacking (55, 56, 96, 97). Therefore, these measures are better regarded as adjunctive options in selected settings rather than replacements for standardized nursing pathways supported by stronger evidence.

Under these challenges, multidisciplinary collaboration is particularly important for the sustained implementation of SSI prevention. Nurses play central roles in pathway coordination, patient education, continuous surveillance, and reporting of abnormalities. Surgeons are responsible for procedure selection, incision management, and decisions regarding infection treatment. Anesthesiologists are responsible for perioperative support of temperature control, glycemic management, and physiological stability. Infection control and microbiology teams are responsible for pathogen surveillance, infection definitions, and feedback on local epidemiology. Clinical pharmacists are responsible for optimizing antimicrobial prophylaxis regimens and monitoring inappropriate antimicrobial use. Information technology teams are responsible for integrating digital tools, alert systems, and follow-up platforms into actual clinical workflows. The value of multidisciplinary collaboration lies not in simply increasing the number of participating departments, but in incorporating preoperative prevention, postoperative recognition, and post-discharge follow-up into a single standardized pathway. Only on this basis can true continuity be established across perioperative prevention, postoperative detection, and out-of-hospital surveillance.

From the perspective of future research and implementation, the priority in SSI prevention should not simply be the addition of more isolated measures, but rather improving implementation quality, standardizing surveillance practice, strengthening the external validation of digital tools, and systematically evaluating clinical outcomes, cost-effectiveness, and patient experience within a multidisciplinary collaborative framework. Future studies should also focus more specifically on which nursing processes most strongly influence adherence to bundled interventions, which digital tools genuinely improve continuity of surveillance, and which quality indicators best reflect stable execution of nursing pathways. Overall, a pathway that is nursing-centered, supported by multidisciplinary collaboration, and sustained by continuous evaluation is more likely to achieve consistent reductions in SSI incidence and improve the quality of perioperative care across different healthcare settings.
